# The Gut Microbiota Is Involved in the Regulation of Cognitive Flexibility in Adolescent BALB/c Mice Exposed to Chronic Physical Stress and a High-Fat Diet

**DOI:** 10.3390/microorganisms12122542

**Published:** 2024-12-10

**Authors:** Cristian Yuriana González, José Antonio Estrada, Rigoberto Oros-Pantoja, María del Carmen Colín-Ferreyra, Alejandra Donaji Benitez-Arciniega, Alexandra Estela Soto Piña, José Félix Aguirre-Garrido

**Affiliations:** 1 School of Medicine, Autonomous University of the State of Mexico, Paseo Tollocan and Jesús Carranza, Toluca de Lerdo 50180, State of Mexico, Mexico; cgonzalez002@alumno.uaemex.mx (C.Y.G.); jaestradag@uaemex.mx (J.A.E.); rorosp@uaemex.mx (R.O.-P.); mdcolinf@uaemex.mx (M.d.C.C.-F.); abeniteza@uaemex.mx (A.D.B.-A.); 2 Department of Biotechnology and Environmental Microbiology, Autonomous Metropolitan University-Lerma, Hidalgo Pte. 46, Lerma 52006, State of Mexico, Mexico; j.aguirre@correo.ler.uam.mx

**Keywords:** attentional set-shifting test, chronic stress, cognitive flexibility, high-fat diet, gut microbiota, prefrontal cortex

## Abstract

Dysfunction in the prefrontal cortex can lead to cognitive inflexibility due to multifactorial causes as included cardiometabolic disorders, stress, inadequate diets, as well as an imbalance of the gut–brain axis microbiota. However, these risk factors have not been evaluated jointly. The purpose of this study was to evaluate the effect of physical stress (MS: Male Stress and FS: Female Stress) and high-fat diet (MD: Male Diet and FD: Female Diet) supplementation on the gut microbiota and cognitive flexibility. Methods: The study was performed on 47 mice, 30 male (M) and 17 female (F) BALBc, exposed to chronic stress physical (S) and high-fat diet (D). Cognitive flexibility was evaluated using the Attentional Set-Shifting Test (ASST) and the gut microbiota composition in terms of relative abundance (%) and alpha–beta diversity. Results: Results showed that S and D reduced cognitive flexibility in male and female mice (*p* < 0.0001). Significant changes occurred in *Alistipes* spp. (MM vs. MS:MD; *p* < 0.0001), *Barnesiella* spp. (FC vs. FS; *p* = 0.0002; FC vs. FD, *p* = 0.0033); *Dorea* spp. (MC vs. MD, *p* = 0.0008; MM vs. MD, *p* < 0.0001) and *Lactobacillus* spp. (MC vs. MD and FM vs. FS, *p* < 0.0001; FM vs. MD, *p* = 0.0393) genera among groups. Predictive functional analysis (QIIME2 and PICRUSt2) showed a significant increase in the expression of histidine kinase, alanine dehydrogenase, glutamine synthase, glutamate synthase, arginine succinyl synthase, and tryptophan synthase genes (*p* < 0.05), the latter being a precursor of serotonin (5-HT). Conclusions: Chronic physical stress and a high-fat diet modify cognitive flexibility and the composition and predictive function of the gut microbiota.

## 1. Introduction

It has been shown that high consumption of saturated fats and chronic exposure to stress can result in cardio-metabolic alterations that consequently lead to cognitive dysfunction [1,2,3,4,5,6]. Moreover, it has been demonstrated how the microbiota–gut–brain axis regulates multiple physiological processes from early ages, highlighting neurodevelopment and its alterations [7,8,9,10,11]. However, the study of this relationship has been complex because there are variables such as diet, drug consumption, lifestyle, and age that affect it. The analysis of alterations in the gut microbiota and the effects this has on neurodevelopment have been poorly studied. Specifically, chronic stress and high dietary intake of carbohydrates and saturated fats have been associated as risk factors for neurological diseases and particularly with cognitive dysfunction [12,13]. Although cognitive flexibility is one of the adaptive functions possibly altered by the gut microbiota abnormalities, and probiotic supplementation in older adults has been shown to improve it, the evidence is still inconclusive [14].

High-fat diets produce hippocampus-dependent cognitive alterations in rodents [15]. Preclinical models have shown that high-fat diets impair cognition independently of weight gain [15]. For example, C57BL/6J mice exhibit hippocampus-dependent cognitive impairment after 1 day of being exposed to a 60% high-fat diet, but cognition improves upon returning to a healthy diet [16]. Interestingly, supplementation with prebiotics could also reverse obesity in a rat model of diet-induced metabolic syndrome [17]. In addition, high-fat diets can alter gut microbiota. Some studies reveal that obesity induced by a high-fat diet is related to an increased ratio of Bacteroidota/Bacillota, which affects the metabolic function of intestinal microbes and increases their energy intake [18,19,20]. Moreover, the gut microbiota can be affected by the exposure to stress [21].

Therefore, it is necessary to understand the bidirectional links between the gut microbiota, cognitive function, diet, and stress and prevent risk factors at early stages of life like adolescence. The analysis of these changes in murine models will facilitate the understanding of this complex association [22]. The aim of this study was to determine whether chronic physical stress and a high-fat diet induce modifications in cognitive flexibility in adolescent BALB/c mice and whether these are related to the structure and composition of the gut microbiota. To do that we used the Attentional Set-Shifting Test (ASST), which is reliable to assess cognitive flexibility [23]. This tool is sensible to detect the stress impact on attention and memory [24], specifically mediated by the prefrontal cortex [2,25,26,27,28].

## 2. Methods

### 2.1. Animals

Male and female BALB/c mice (*n* = 47), 10 weeks of age, were housed at the Animal Facilities of the School of Medicine of the Autonomous University of the State of Mexico. The animals were housed in polycarbonate cages. Food (Lab Diet, Laboratory Rodent Diet, Catalog 5001, USA) and water were given ad libitum. Mice were maintained under a 12–12 h light-dark cycle, at 20–24 °C room temperature and 45–55% relative humidity. The whole experimental procedure lasted 21 days. For bioethical reasons and a review of international guidelines, a minimum of 6 mice for each group of males and 4 mice for each group of females were included. Experimental groups were as follows: male control (MC), female control (FC)*,* male high-fat diet (MD), female high-fat diet (FD), male stress (MS), and female stress (FS), n = 8–9 males and 4–5 females per group. In addition, two additional manipulation control groups were included to compare the gut microbiota in males (MM) n = 5 and females (FM) n = 4. The control, stress, and high-fat diet groups were subjected to food restriction to 70% of their Recommended Daily Intake (RDI, 5 g/day) for the ASST evaluation. Body weight was measured 3 times per week on non-food restriction days and daily on food restriction days, prior to the cognitive function tests. Mice with a weight loss >20% were excluded from the cognitive test. The animals were kept under the conditions established by the Mexican Animal Guidelines (NOM-062-ZOO-1999) [29] with registration data CONBIOETICA-15-CEI-002-20210531 approved by Comisión Nacional de Bioética, UAEMéx.

### 2.2. Exposure to Chronic Physical Stress (MS and FS Groups)

All mice were given food and water ad libitum prior to exposure to S. This consisted of exposing mice to 4 °C for 30 min during 14 consecutive days [30], prior to cognitive testing. Animals were placed inside a circular container cooled with ice at 4 °C. The container had small holes around the edge to allow mice proper breathing, as a glass lid was used to prevent the mice from escaping out of the container.

### 2.3. Exposure to High-Fat Diet (MD and FD Groups)

Animals were fed with the standard rodent diet (Lab Diet^®^, Laboratory Rodent Diet 5001). For the high-fat diet groups, the same diet a were weighed and mixed with distilled water and 20% commercial saturated fat (lard) [31]. From this resulting paste, the pellets were molded, drying, cooling, and storage at 4°C. All mice were given water ad libitum throughout the procedure.

### 2.4. Attentional Set-Shifting Test (ASST)

Mice were placed in an acrylic ASST chamber measuring (30.48 × 20.32 × 17. 78 cm). This chamber consists of two areas: an initial or waiting area and a test or decision-making area. In the initial area, a bowl with water was placed, and in the test area, two bowls were placed for decision-making by the mice (one of them with a reward). As a reward stimulus, mice were given cereal honey-flavored Cheerios^®^ (Nestlé, Vevey, Switzerland) (20–30 mg each, approximately). Mice were given 3 min to perform the trials correctly. If a mouse did not make a choice within 3 min, the trial would be considered failed, and if it did not conclude any of the trials, the animal would be excluded from the study. The ASST was performed as described in Heisler et al. (2015) [2]: On day 1, animal manipulation and body weight recording started and continued until the end of the experiment. From day 9 to day 17, food restriction at 70% of mice RDI (5 g/day), and acclimatization to the test room began simultaneously. On days 13 and 14, acclimatization to the ASST chamber started, so the mice could become familiar with it. On day 15, training was performed. This is one of the most important days because the mice learned how to dig and find the reward within the digging medium (sawdust); if they failed to learn that on the scheduled day, a second day of training had to be scheduled. On day 16, 3 tasks were performed: simple dimension (SD), compound dimension (CD), and the first reversal (R1), and on day 17, the remaining 4 trials were carried out: interdimensional shift (IDC), second reversal (R2), extradimensional shift (EDC), and third reversal (R3). The tests were considered successful once 8 successful trials were achieved continuously, with no errors in the mice’s decisions made in response to the stimuli.

### 2.5. Microbiome Sample Collection and Metagenomic DNA Extraction

After ASST evaluations were completed, the gut microbiota was collected, and all the mice were euthanized in a CO_2_ chamber for subsequent removal of the colon. For the analysis of the gut microbiome, samples were taken from the contents of the colon of mice. The colon was dissected out and washed with Phosphate-Buffered Saline (PBS) at pH 8.0. The contents in PBS were stored at −80 °C for further analysis. Metagenomic DNA was extracted from the samples using the Quick-DNA Fecal/Soil Microbe Miniprep Kit (Zymo Research, Irvine, CA, USA). The quality of extracted DNA was determined by band analysis in 1% agarose gel electrophoresis. DNA concentration and purity were measured by spectrophotometry (A260/280 and A260/230 nm). The metagenomic DNA samples obtained were stored at −20 °C.

### 2.6. Sequencing and Bioinformatics Analysis

The V3–V4 region of the 16S rRNA gene was amplified using recombinant Taq DNA polymerase (Thermo Scientific, Waltham, MA, USA). After that, sequencing was performed using the MiSeq System—Illumina sequencing platform (2 × 300 bp) using 341F/805R primers [32] [Integrated Microbiome Resource (IMR), https://imr.bio (accessed on 20 September 2024), Halifax, Nova Scotia, Canada]. The sequences generated by IMR were processed and analyzed using the bioinformatics software Mothur v.1.48.0 [http://mothur.org (accessed on 20 September 2024)] [33]. Then, the sequences were filtered with quality scores > 25%, length < 75%, and chimeric sequences were eliminated with the chimera.vsearch command [34]. The non-chimeric sequences were assigned as operational taxonomic units (OTUs) with 97–98% taxonomic identity to the SILVA-base bacterial reference alignment [https://www.arb-silva.de/ (accessed 21 November 2024)] for the taxonomic identification of bacteria [35]. A maximum length of 8 homopolymers and 0 ambiguities was established. Finally, a Mothur-formatted version of the RDP training set (v.9) was used to complete the alignment with identities ≥ 97% [36]. Eukaryotic taxa and archaea were excluded using the Mothur taxon filter [37]. Each sample calculated the alpha diversity indices with richness (Chao1) and diversity (Shannon–Wiener and inverse Simpson) calculated in each sample. Beta diversity was performed with a principal component analysis (PCA) on each sample to observe the trend of the experimental groups (PC1 x-axis and PC2 y-axis). Alpha and beta diversity were used to differentiate the bacterial communities’ structure in each sample using Statistical Analysis of Metagenomic Profiles Bioinformatics (STAMP Bioinformatics) software v. 2.1.3. (*p* < 0.05) [38,39].

### 2.7. Taxonomic Identification and Prediction of Functional Gene Content

Taxonomic identification sequencing data were submitted to the National Center for Biotechnology Information (NCBI) platform with access data to BioProject PRJNA1183986 (https://www.ncbi.nlm.nih.gov/sra/PRJNA1183986 accessed on 9 November 2024). To assess the metabolic potential of microbial communities found in experimental groups, 16S rRNA sequence reads were clustered into operational taxonomic units (OTUs) using the closed-reference method in QIIME2 v.2022.2 software [40]. The generated OTU table was imported into the PICRUSt2 v.2.4.15 [41] and the Kyoto Encyclopedia of Genes, and the Genomes (KEGG) [42] database was used to predict the functional gene content of the various microbial communities represented in the Green Genes database for 16S rRNA gene sequences [43,44]. The output from the KEGG database containing the predicted function was further analyzed using linear discriminant analysis (LDA) combined with linear discriminant analysis effect size (LEfSe). The purpose of this was to identify the most abundant predictions in the different experimental groups [45]. The predicted functions output from KEGG was also exported to STAMP version 2.1.3 for further statistical analysis and plotting.

### 2.8. Statistical Analysis

For the analysis of the data obtained in the ASST, the maximum number of criterion trials performed in each of the ASST stages for each mouse was recorded [data were presented as mean ± standard error (S.E.) values]. Shapiro–Wilk and Kolmogorov–Smirnov normality tests were performed to verify that the data complied with the normality assumption. Two-way ANOVAs were used to compare significance among experimental groups. Statistical significance was considered with a value of *p* < 0.05. Tukey’s post hoc tests were performed for the multiple comparisons of means between groups. The data analyzed for microbial composition were expressed as relative frequency percentages (%). Student’s *t*-tests were performed for comparison of independent groups for comparative analysis of the abundance of the microbiota. Two-way ANOVA was used to compare significance among experimental groups for alpha diversity (*p* < 0.05). For beta diversity PCA, Software Statistical Analysis of Metagenomic Profiles Bioinformatics (STAMP Bioinformatics v. 2.1.3) was used; the same software was used for the comparative ANOVA of the experimental groups in the functional analysis. All the analyses were performed using GraphPad Prism version 9 (Dotmatics; Woburn, MA, USA) and STAMP Bioinformatics. A statistically significant level of *p* < 0.05 was considered for these tests.

## 3. Results

### 3.1. Attentional Set-Shifting Test in Groups Stress (MS/FS) and High-Fat Diet (MD/FD)

**Figure 1:**
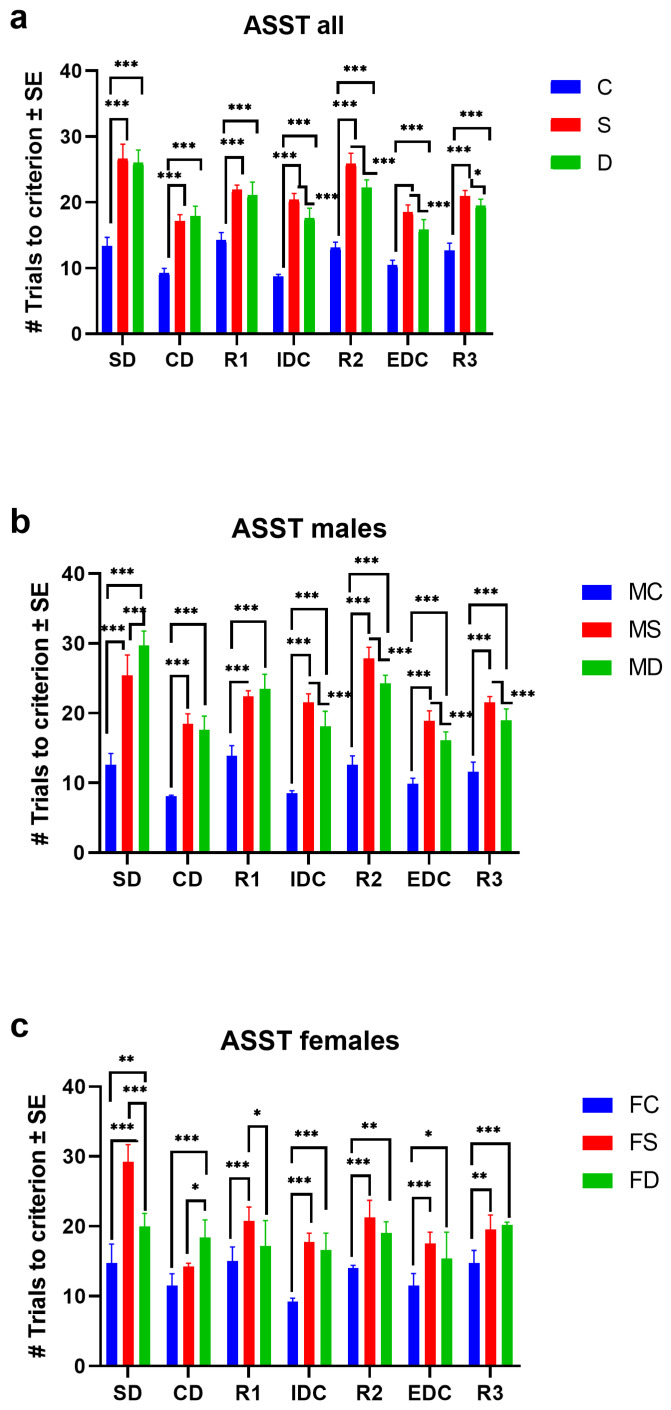
Attentional Set-Shifting Test (ASST) results for male and female BALB/c mice. (**a**) ASST results for the entire mouse population (n = 38) in control (C), chronic physical stress (S), and high-fat diet (D) for male (M) and female (F) mice *(F*_12, 245_ *=* 17.08, *p* < 0.0001). (**b**) ASST results for male mice. The number of trials to criterion shows significant differences in the stress and the high-fat diet vs. the control group (n = 8–9 animals per group) (*F*_12, 154_ = 15.67, *p* < 0.0001); (**c**) ASST results for female mice (*F*_12, 70_ = 5.232, *p* < 0.0001). The number of trials to criterion shows significant differences in the stress and high-fat diet group vs. the control group (n = 4–5 animals per group); Two-way ANOVA with significance level *p*-value * *p* < 0.1, ** *p* < 0.01, *** *p* < 0.001. Data are shown as mean (number of criterion trials) ± standard error (SE); control (C), stress (S), diet (D), male control (MC), male stress (MS), male diet (MD), female control (FC), female stress (FS), female diet (FD), simple discrimination (SD), compound discrimination (CD), reversion 1 (R1), interdimensional changes (IDC), reversion 2 (R2), extradimensional changes (EDC), and reversion 3 (R3).

The results obtained for the ASST in the two-way ANOVA showed an increase in the number of trials to criterion significant in the interaction of all groups [Fisher distribution and degrees of freedom numerator/denominator, F(_DFn, DFd_) = F_12, 245_ = 17.08, *p* < 0.0001]. Tukey’s multiple comparisons test was significant for the stress and diet groups vs. the control group in SD, CD, R1, IDC, R2, EDC, and R3 (*p* < 0.0001). The stress vs. diet groups were significantly different in the IDC, R2, EDC, and R3 stages (*p* < 0.0001). For the SD (*p* = 0.4504), CD (*p* = 0.2935), and R1 (*p* = 0.2331) stages, there were no significant differences (Figure 1a). Data mean ± S.E. and *p*-values are shown in the Supplementary Material (Table S1).

For the group of male mice, the two-way ANOVA interaction of the groups showed a significant increase in the number of trials to criterion (F_12, 154_ = 15.67, *p* < 0.0001). The MD and MS groups had a significantly higher number of trials than the control group in all the ASST stages (*p* < 0.0001). There were no significant differences for the comparison between the stress vs. diet groups in the CD (*p* = 0.5241) and R1 (*p* = 0.3325) stages (Figure 1b).

The two-way ANOVA interaction of the female mice groups was significant, showing an increase in the number of criterion trials performed in the ASST in females exposed to stress and a high-fat diet (F_12, 70_ = 5.232, *p* < 0.0001). Tukey’s multiple comparisons test revealed significant differences in the FC group vs. FS in SD (*p* < 0.0001), R1 (*p* = 0.0009), IDC (*p* < 0.0001), R2 (*p* < 0.0001), ECD (*p* = 0.0005), and R3 (*p* = 0.0069). There were no significant differences in the CD (*p* = 0.1707) stage. The number of trials in the FD group was significantly higher than that of the control group in the SD (*p* = 0.0014), CD (*p* < 0.0001), IDC (*p* < 0.0001), R2 (*p* < 0.0024), EDC (*p* = 0.0222), and R3 (*p* = 0.0009) stages. There were no significant differences in the R1 (*p* = 0.2813) stage. For the comparison between the FS vs. FD groups, there were significant differences in the SD (*p* = 0.0001), CD (*p* = 0.0139), and R1 (*p* = 0.0411) stages. However, there were no significant differences for the IDC, R2, EDC, and R3 stages (Figure 1c). The *p*-values of the multiple comparisons in the Tukey test of the experimental groups are shown in Supplementary Table S2.

### 3.2. Analysis of Alpha and Beta Diversity Indices

**Figure 2:**
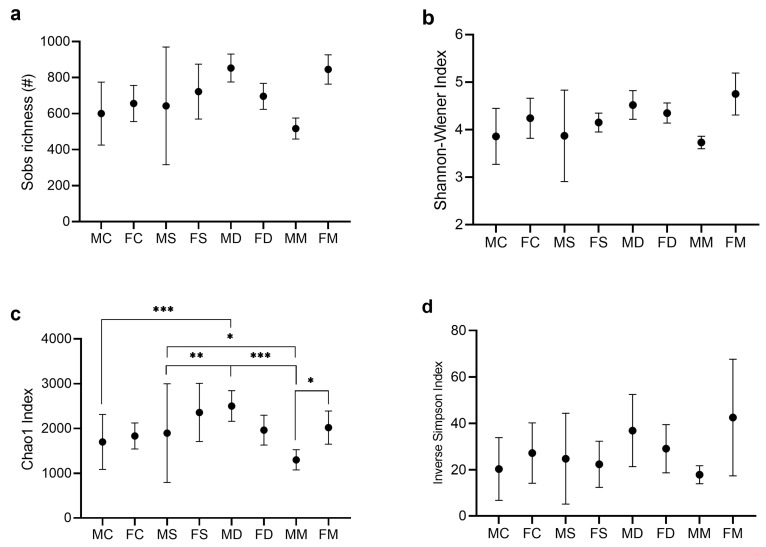
Alpha diversity indices experimental groups. Alpha diversity is depicted as (**a**) Sobs Richness number, (**b**) Shannon–Wiener, (**c**) Chao1, and (**d**) inverse Simpson Indices. * Two-way ANOVA with significance level *p*-value < 0.05; *** *p* = 0.0004, ** *p* = 0.0059, * *p* = 0.0318, *** *p* < 0.001, * *p* < 0.0311. Only the significant *p*-values are shown in the figure. Data shows the gut microbiome richness and diversity indices as mean ± low and high confidence intervals (LCI-HCI). MC, male control; FC, female control; MS, male stress; FS, female stress; MD, male diet; FD, female diet; MM, male manipulation; and FM, female manipulation.

Because diversity indices are sensitive to the number of sequences, diversity was delimited based on the sample containing the lowest number of minor sequences, i.e., 13,291. Coverage ranged from 94% to 99% in terms of the number of OTUs. Alpha diversity included Sobs (richness rate), the Chao1 index (richness), the Shannon–Wiener index (diversity), and inverse Simpson indices (Figure 2a–d). No significant differences were seen among all groups for Sobs, Shannon, and inverse Simpson index. However, for the Chao index, group MC vs. MD (*p* = 0.0004), MS vs. MD (*p* = 0.0059), MS vs. MM (*p* = 0.0318), and MD vs. MM (*p* < 0.0001) were significant in male mice (Figure 2c), indicating higher microbial species richness in the groups exposed to stress and diet vs. the control and manipulation control groups. Moreover, there were no significant differences in female groups. Significant differences were also found between males and females in MM vs. FM groups (*p* = 0.0311).

**Figure 3:**
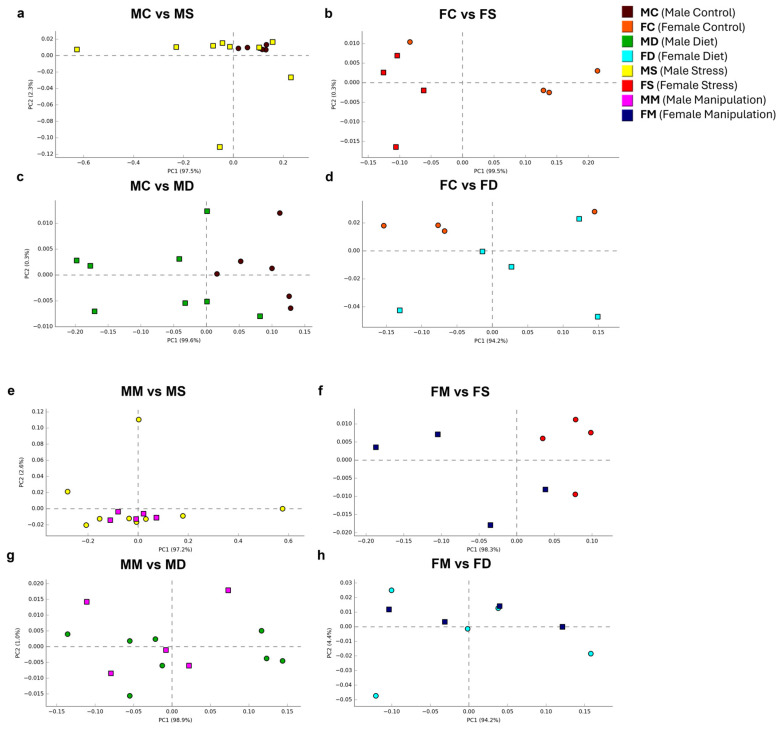
Beta diversity indices in experimental groups. Beta diversity at the phylum level is based on principal component analysis (PC1 x-axis and PC2 y-axis) of grouped variables expressed as a percentage (%) PC1 96.5% vs. PC2 2.2% in all groups. Comparative analysis of beta diversity between groups: (**a**) PC1 97.5% vs. PC2 2.3% (MS vs. MC), (**b**) PC1 99.5% vs. PC2 0.3% (FS vs. FC), (**c**) PC1 99.6% vs. PC2 0.3% (MD vs. MC), (**d**) PC1 94.2% vs. PC2 5.4% (FD vs. FC), (**e**) PC1 97.2% vs. PC2 2.6% (MS vs. MM), (**f**) PC1 98.3% vs. PC2 1.0% (FS vs. FM), (**g**) PC1 98.9% vs. PC2 1.0% (MD vs. MM), and (**h**) PC1 94.2% vs. PC2 4.4% (FD vs. FM). MC: male control (brown); FC: female control (orange); MS: male stress (yellow); FS: female stress (red); MD: male diet (green); FD: female diet (light blue); MM: male manipulation (light purple); and FM: female manipulation (dark blue).

Beta diversity calculated at the phylum taxonomic level by the group indicated that the PC of the male control (MC) group showed a trend towards the right quadrant (PC1) representing 97.6% of the grouped variables, while the female control (FC) group showed a tendency towards the left (PC1 95.8%). The PC1 in the MS and MD groups showed a shift to the left from the MC group (PC1 97.5%, 99.6%, respectively, Figure 3a,c). The PC1 in the MM group shifted to the left in groups MS and MD (PC1 97.2%, 98.9%, respectively, Figure 3e,g). The PC1 axis showed that with respect to the FC group, the principal component shifted to the left in the FS group (PC1 99.5%, Figure 3c) or to the right in the FD group (PC1 94.5%, Figure 3d). With respect to the FM group, the PC1 was shifted to the right for the FS group (PC1 98.3%, Figure 3f) or to the left for the FD group (PC1 94.2%, Figure 3h). These results indicate that the PC1 axis evidenced well-marked trends and notable differences between males and females subjected to chronic physical stress and a high-fat diet.

### 3.3. Microbial Composition

**Figure 4:**
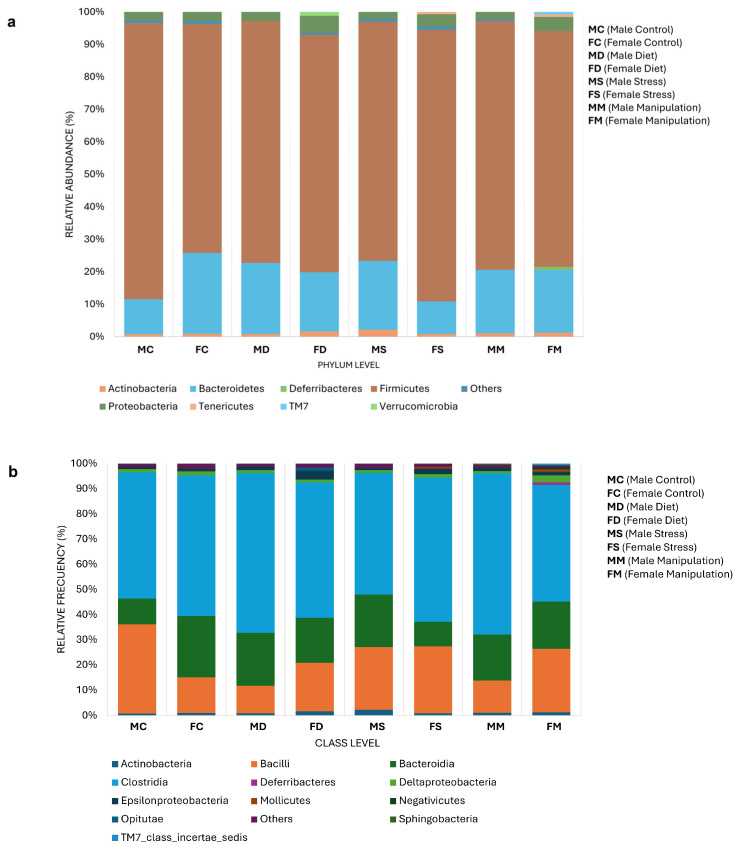
Relative abundance of microbial taxa in experimental groups. (**a**) Phylum and (**b**) class taxa in male control (MC), male stress (MS), male diet (MD), male manipulation (MM), female control (FM), female stress (FS), female diet (FD), and female manipulation (FM) groups. Relative abundance expressed in %. MC: male control; FC: female control; MD: male diet; FD: female diet; MS: male stress; FS: female stress; MM: male manipulation; and FM: female manipulation.

Sequencing results showed the taxonomic levels of Bacteroidota and Bacillota at the phylum level as the most abundant in the microbiome of male mice. The phylum Bacteroidota varied in abundance from 19% to 21% in the stress group and 22% in the high-fat diet group. On the other hand, the phylum Bacillota varied from 77% to 74% for the stress group and from 77% to 73% for the diet group. For female mice, sequencing results also show Bacteroidota and Bacillota as the most abundant phyla. For the phylum Bacteroidota, the abundance varied from 19% to 9% in the stress group and 18% in the diet group. For Bacillota, it ranged from 72% to 73% in the stress group and 83% in the diet group (Figure 4a).

At the class level, the abundance was most prominent in Clostridia, followed by Bacilli and Bacteroidia; Actinomycetes, Deltaproteobacteria, and Epsilonproteobacteria had lower abundance; negligible abundance of some families was seen in the MC/FC and MM/FM groups (Figure 4b). Regarding the control and manipulation groups, the abundance of Clostridia increased in the FS and FD groups and then decreased in the MS and MD groups. In the case of Bacilli, the abundance decreased in the high-fat diet groups (MD and FD) and increased in the stress groups (MS and FS). For the Bacteroidia class, there was an increase in abundance in the male mice (MS and MD) and a decrease in the female groups (FS and FD) (Figure 4b).

### 3.4. Comparative Microbial Analysis

**Figure 5:**
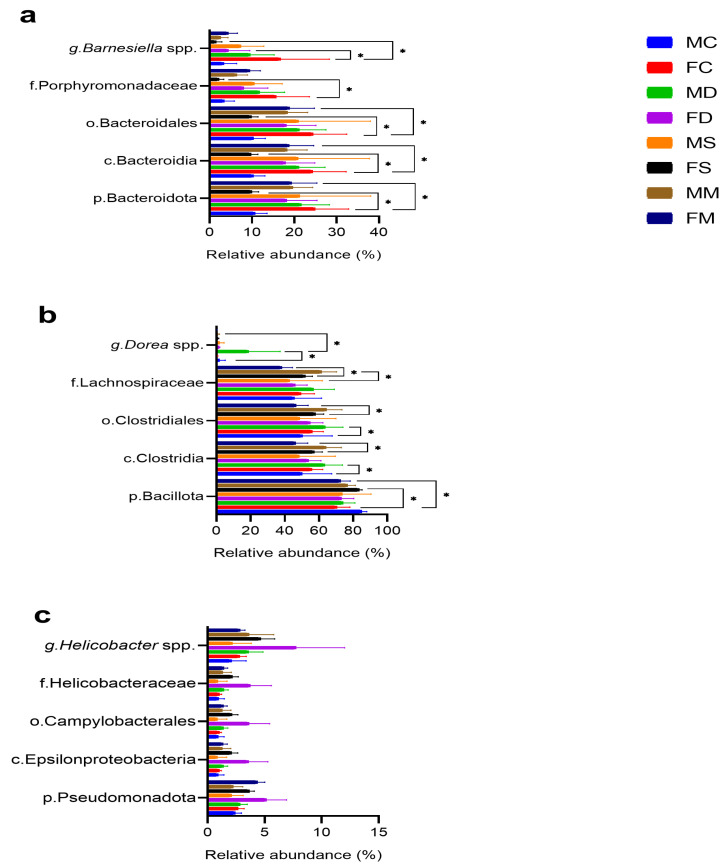
Comparative microbial analysis in experimental groups. Comparison among the phyla (**a**) Bacteroidota, (**b**) Bacillota, and (**c**) Pseudomonadota at class (c.), order (o.), family (f.), and genus (g.) levels of relative abundance respective to the male control (MC) in stress (MS), diet (MD), and manipulation (MM) groups, as well as female ones: control (FC), stress (FS), diet (FD), and manipulation (FM). Multiple *t*-tests with significance *p* < 0.05. Only significant values are represented in the figure * p < 0.05. Please see *p*-values in Table S3. Groups are labeled as follows: MC: male control; FC: female control; MS: male stress; FS: female stress; MD: male diet; FD: female diet; MM: male manipulation; and FM: female manipulation.

A comparative analysis was performed among the phyla Bacteroidota, Bacillota, and Pseudomonadota (Figure 5). Significant changes were observed in the various lineages compared with observed increases in Bacteroidota and decreases or increases for Bacillota. The decrease in Bacteroidota or increase in Bacillota was found only in the FS vs. FC and FM groups (*p* < 0.01) vs. with the other groups: MC vs. MS, MC vs. MD, FC vs. FD, MM vs. MS, MM vs. MD, and FM vs. FD (Table S3).

### 3.5. Comparative Prediction of Functional Gene Content Analysis

**Figure 6:**
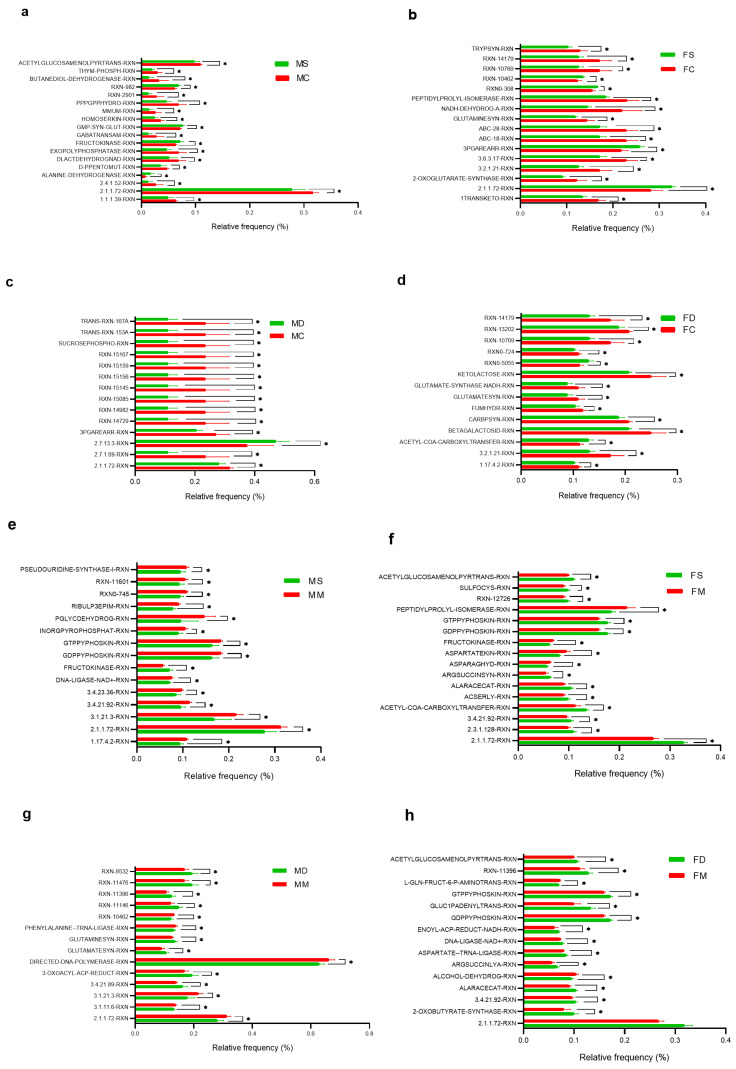
Predictive comparison of metabolic pathways associated with the gut microbiome in experimental groups. (**a**) Male control (MC) vs. male stress (MS), (**b**) female control (FC) vs. female stress (FS), (**c**) male control (MC) vs. male diet (MD), (**d**) female control (FC) vs. female diet (FD), (**e**) male manipulation (MM) vs. male stress (MS), (**f**) female manipulation (FM) vs. female stress (FS), (**g**) male manipulation (MM) vs. male diet (MD), and (**h**) female manipulation (FM) vs. female diet (FD). Relative frequency expressed in %. * *p*-value < 0.05. RXN, reaction.

Prediction of functional gene content analysis was performed for MC:FC and MM:FM groups vs. exposure to chronic stress physical (MS and FS) and high-fat diet (MD and FD) for both male and female mice. The main significant enzymes for the different groups, which could regulate the different metabolic pathways, are shown predictively (*p* < 0.05). In most groups, pathways related to lipid metabolism (fatty acids and triglycerides), carbohydrates (glucose and fructose), proteins (amino acids), nucleic acids (DNA methylation), and the Krebs cycle (Figure 6).

The increased enzymatic pathways found in the different treated groups are glutathione reductase, GMP-Synthase–GLUT, fructokinase, and alanine dehydrogenase (MS vs. MC); histidine kinase (MD vs. MC); DNA methyltransferase, phosphoglycerate mutase, L-cysteine sulfotransferase desulfurase, and glycerate–acyltransferase (FS vs. FC); acetyl-CoA carboxyl transferase, biotin carboxyl, beta-galactosidase, beta-glucosidase, and ketolactose (FD vs. FC); fructokinase (MS vs. MM); oxo-glutaryl methyl ester reductase, fatty acid synthase, oxo-dGTP diphosphatase, glutamine synthase, glutamate synthase, and peptidase I (MD vs. MM); DNA methyltransferase, acetyl-CoA carboxyl transferase, acetyl serine sulfyhidrathase, alanine racemase, arginine succinyl synthase, and tryptophan synthase (FS vs. FM); and DNA methyltransferase, gluc1panedyl transferase, enoyl-ACP reductase, DNA ligase, aspartate–tRNA ligase, arginine succinyl lyase, alanine racemase, endopeptidase, and 2-Oxobutyrate synthase (FD vs. FM) (*p* < 0.05).

## 4. Discussion

In this study, we found that chronic physical stress and high-fat diet affect ASST performance in the SD, CD, R1, IDC, R2, EDC, and R3 stages, which indicates that these promote cognitive impairment in adolescents. This agrees with the previous results in rodents [46,47,48]. Yu et al., 2021, evaluated the effect of the high-fat diet in BALBc mice with depression, finding that the high-fat diet could promote a depressive type of behavior because blood lipid concentrations were significantly elevated in this group, generating an uncontrolled lipid metabolism that accelerated the depressive process [49]. Nonetheless, a limitation of this study is the lack of measurements of plasma concentrations of lipids, enzymes, or neurohormones that could have a bidirectional relation within the microbiota–gut–brain axis.

In the ASST results, the high-fat and stress groups present a higher number of trials to criterion in the IDC and EDC stages than controls. This is consistent with studies in rats reporting that cognitive inflexibility occurs mainly in the IDC/EDC and reversal changes in ASST [4,50]. However, the reversal learning (stages R1, R2, and R3) is more affected than IDC/EDC in both male and female mice; this suggests a greater negative impact on reversal learning in adolescent mice subjected to chronic physical stress and a high-fat diet. This is similar to other experiments where ASST performance is altered by various types of stressors and a high-fat diet [27,51,52,53].

Our investigation also reveals some difference in cognitive flexibility by sex. For example, stressed females show no significant increase in CD stage, while females on a high-fat diet show no significant increase in R1 stage. Males show a significant increase in both stages (CD and R1). This could indicate a greater cognitive flexibility in females than in males in compound and reversal learning. Also, we found a significant increase in IDC, R2, EDC, and R3 stages in males and in CD and R1 stages in females. This suggests that chronic physical stress and high-fat diets mostly affect IDC/EDC in males and compound learning in females. Reversal learning is affected in both males (R2 and R3) and females (R1). This is in agreement with previous studies that have found greater involvement in IDC, EDC, and reversion stages in male rodents [27,50]. Nonetheless, this is the first study that evaluates cognitive flexibility in females, so this precedent is left for further research in female mice.

In humans, alterations in PFC functions are related to the development of cognitive impairment [54]. Neurovascular alterations are associated with cognitive dysfunction and stress in adolescence [55]. Sustained chronic stress for prolonged periods of time (from months to years) produces an excess secretion of glucocorticoids like cortisol that can produce hippocampal atrophy [56]. Besides the PFC, the hippocampus is another essential structure for learning and memory that has a high concentration of glucocorticoid receptors [57], and this is associated with the PFC [58]. In addition, norepinephrine and epinephrine released under stress are associated with cognitive performance in adolescents [59]. Previous behavioral research has shown that adolescent executive function is different from that of adults [4]. For example, adolescent rats have an increased number of criterion trials when performing IDC, EDC, and reverse learning tests, thus showing greater cognitive rigidity than adults [4]. Therefore, the findings found in BALBc adolescent mice with chronic physical stress and a high-fat diet could help prevent or delay early-stage damage to the PFC.

Studies have shown that exposure to chronic stress, physical activity, and high-fat diets also affects the abundance of the gut microbiota in young mice [60,61]. We found altered microbial alpha diversity in males subjected to stress (MS vs. MM and MD) and high-fat diets (MD vs. MC and MM) compared to controls. There were also significant differences in the control groups of male vs. female (MM vs. FM). In terms of beta diversity, the results show defined group trends to the right or left for males and females subjected to chronic stress, physical activity, and a high-fat diet. This would indicate a greater richness of microbial species in females vs. males exposed to chronic stress, physical activity, and a high-fat diet. This coincides with that reported by Yanguas-Casás et al., 2021, who found that a high-fat diet in mice under chronic restraint stress in a model of Alzheimer’s disease induced gut dysbiosis, which translated into a reduction in microbiota diversity and abundance as well as an increased expression of inflammatory genes in male and female mice [60].

Regarding the composition of the microbiota, we found the phyla Bacteroidota and Bacillota (Pseudomonadota to a lesser extent) are the most abundant in all groups. This coincides with other reports [62,63,64,65,66]. In this investigation, males with chronic stress, physical activity, and a high-fat diet show an increase in the percentage of relative abundance in Bacteroidota and a decrease in Bacillota. However, in females the opposite occurred, with a decrease in Bacteroidota and an increase in Bacillota. In the literature, consumption of a high-fat diet is generally associated with a decrease in Bacteroidota and an increase in Bacillota; these alterations in the composition of the gut microbiota have been associated with obesity and the subsequent development of chronic diseases [67]. In this study, a decrease in Bacteroidota and an increase in Bacillota are found only in the FS group vs. controls (FC and FM) (*p* < 0.001). Nonetheless, 8-week-old female C57BL/6J mice exposed to chronic electric shock-induced stress show significant changes in the proportion of the Lactobacillaceae family belonging to the phylum Bacillota [68]. Alterations in this stress group are associated with changes in cytokines related to eosinophilic immune activity [69]. This suggests that this type of change in the gut microbiota could produce dysbiosis as it occurs in women, leading to many diseases [70,71].

We found that *Dorea* spp. genus significantly increases in males with a high-fat diet and a decrease in the relative abundance of the Lactobacillus genera. Similarly, adolescent male rats supplemented with a high-fat diet for 9 weeks showed an increase in the *Dorea* spp. genus and a decrease in Lactobacillus genera [53]. Moreover, in a study conducted on high-fat diet-induced obese mice, the supplementation of *Lactobacillus plantarum* FRT4 reversed obesity by modulating the gut microbiota and liver metabolome [72]. Similarly, in our study, a high-fat diet significantly affects the composition of Lactobacillus spp. genera in male mice. In females, we found that the Lactobacillus genera also presented significant changes, increasing in females with chronic stress and physical activity and decreasing in females with a high-fat diet. However, a study in adolescent C57BL/6J mice (male and female, 6–8 weeks old) subjected to water avoidance stress (WAS) [68] produces activation of the hypothalamic–pituitary–adrenal (HPA) axis, but there are no significant differences in the Lactobacillus genera in female stress vs. controls. The modification of the microbiota composition correlated with the activation of the HPA axis, which could indicate changes in behavior [68] and PFC.

In the results obtained in the prediction model analyzed (QUIIME2/PICRUSt2), we found enzymes that could be part of some metabolic pathways or routes that could be part of the gut–brain axis. We found in males with a high-fat diet a significant increase in the enzyme histidine kinase (MS vs. MC). In humans, histidine catabolism affects the microbiota composition in diseases such as obesity [73,74] and type 2 diabetes mellitus [75]. A study reported that excess β-alanyl-L-histidine produces memory deficits in Alzheimer’s disease transgenic mice fed with a high-fat diet [76]. A high-fat diet reduces vagal sensitivity to gut mediators such as serotonin and cholecystokinin (CCK); in addition, gut-to-brain communication is mediated by short-chain fatty acids (SCFA) and gut inflammation [77]. Another finding we found in the predictive model was enzymes related to lipid metabolism, such as fatty acid synthase, in male mice exposed to a high-fat diet (MD vs. MM). Studies in rodents show that a relative deficiency of SCFA or SCFA-producing bacterial species observed after chronic feeding of high-fat diets can produce neuroinflammation [65,66]. This is related to increased microglial activity that promotes synaptic remodeling and neurodegeneration [78,79]. We also found that some enzymes that regulate the metabolism of important amino acids such as glutamine, phenylalanine, asparagine, arginine, alanine, and tryptophan are altered in male and female mice with chronic physical stress and high-fat diets. The interaction between the gut microbiota and the brain is also mediated by amino acid-derived metabolites [80]. An example is a study in pregnant female mice that shows an increase in the metabolic pathway of glutamate synthesis after supplementation with a high-fat diet [81]. The results of our predictive model indicate a significant alteration in lipid and amino acid metabolism in male and female mice exposed to chronic physical stress and a high-fat diet.

Another potential mechanism of communication between the gut and the brain is the metabolism of tryptophan (Trp) [82]. Preclinical studies indicate that alterations in the gut microbiota following exposure to chronic stress may be related to the development of anxiety and depression [83]. Synthesis of the neurotransmitter serotonin is tryptophan-dependent; in mice, the absence of microbiota in early life leads to increased plasma tryptophan concentrations and hippocampal serotonin levels in adulthood [82]. Mice with sustained chronic stress develop depression-like behavior and anxiety as well as alteration in the tryptophan metabolic pathway [84]. In addition, kynurenine (Kyn) and its metabolites, an important metabolic pathway of Trp, are strongly activated in the brain [85]. This activation could be induced or modulated by cytokines such as IFN-α and IFN-γ [86]. Interestingly, the toxic signaling of Kyn was mainly observed in the gut, especially in the colon. These results suggest that long-term stress alters Kyn metabolism and endocrine function in the gut–brain axis, together with altered homeostasis of certain microbiota, collectively contributing to the development of depressive behavior [87]. Resulting in a probable predictive pathway of communication between the microbiota–gut–brain axis in females subjected to chronic physical stress.

The most important strength of this research is that it is the first study that shows the impact of chronic physical stress and high-fat diet exposure on cognitive flexibility as well as the composition and function of the gut microbiota in adolescent mice. Therefore, the analysis of the possible metabolic pathways represented in this study is predictive in nature; however, they coincide with some reports in the literature. Significant changes in the microbiota have a greater impact on males subjected to a high-fat diet and on females subjected to chronic physical stress. One of the main limitations is that the microbiota–gut–brain relationship was evaluated through a predictive model. Future studies are required to evaluate certain biochemical and neurochemical parameters that could demonstrate more precisely the regulatory mechanisms that could be participating in this axis.

## 5. Conclusions

Exposure to chronic physical stress and a high-fat diet induces cognitive flexibility alterations in BALBc mice and changes the microbiota abundance. Especially, there were modifications in the Bacteroidota and Bacillota phyla and *Alistipes* spp., *Barnesiella* spp., *Dorea* spp., and *Lactobacillus* spp. genera. It also affects alpha and beta diversity, mostly in males subjected to a high-fat diet and females subjected to chronic physical stress. The main metabolic pathways that could be involved in the gut–brain axis are the ones related to the metabolism of lipids and amino acids. Therefore, future investigations are required to prevent and reverse the effects of chronic stress and high-fat diets in the microbiota–gut–brain axis.

## Data Availability

The original contributions presented in the study are included in the article/Supplementary Material, further inquiries can be directed to the corresponding author.

## Supplementary Materials

The following supporting information can be downloaded at: https://www.mdpi.com/article/10.3390/microorganisms12122542/s1, Table S1: Effects of chronic physical stress and high-fat diet on AST test performance; Table S2: Tukey’s multiple comparisons test on stress and diet groups; Table S3: Multiple *t*-test comparative analysis of taxon abundance among groups.
