# Supplementary material for: The Gut Microbiota Is Involved in the Regulation of Cognitive Flexibility in Adolescent BALB/c Mice Exposed to Chronic Physical Stress and a High-Fat Diet
Source: Microorganisms. 2024 Dec 10;12(12):2542. doi: 10.3390/microorganisms12122542 (PMC11677384; doi:10.3390/microorganisms12122542)
Supplement: Supplementary file 1 [file microorganisms-12-02542-s001.zip › microorganisms-3263453-supplementary.pdf]

Supplementary Table S1. Effects of chronic physical stress and high-fat diet on AST test performance

	Groups					
Stages	**MC (n=8)**	**FC (n=4)**	**MS (n=9)**	**FS (n=4)**	**MD (n=8)**	**FD (n=5)**
Simple discrimination	12.6 ± 1.5	14.7 ± 2.6	25.4 ± 2.9	29.2 ± 2.4	29.7 ± 2.0	20.0 ± 1.8
Compound discrimination	8.1 ± 0.1	11.5 ± 1.7	18.4 ± 1.4	14.2 ± 0.4	17.6 ± 1.9	18.4 ± 2.5
Reversal 1	13.8 ± 1.4	15.0 ± 2.0	22.4 ± 0.7	20.7 ± 2.0	23.5 ± 2.0	17.2 ± 3.6
Intradimensional shift	8.5 ± 0.3	9.2 ± 0.4	21.5 ± 1.2	17.7 ± 1.2	18.1 ± 2.1	16.6 ± 2.4
Reversal 2	12.6 ± 1.2	14.0 ± 0.4	27.8 ± 1.5	21.2 ± 2.4	24.2 ± 1.1	19.0 ± 1.6
Extradimensional shift	9.8 ± 0.8	11.5 ± 1.7	18.8 ± 1.4	17.5 ± 1.6	16.1 ± 1.1	15.4 ± 3.7
Reversal 3	11.6 ± 1.3	14.7 ± 1.7	21.5 ± 0.8	19.5 ± 2.1	19.0 ± 1.6	20.2 ± 0.3

Data are expressed as mean ± standard error. MC: Male Control, FC: Female Control, MS: Male Stress, FS: Female Stress, MD: Male Diet, FD: Female Diet.

Supplementary Table S2. Tukey´s multiple comparisons test on stress and diet groups.

	All mice		Male mice		Female mice	
Group comparisons	**P value**	**Summary**	**P value**	**Summary**	**P value**	**Summary**
SD						
Control vs Stress	<0.0001	****	<0.0001	****	<0.0001	****
Control vs Diet	<0.0001	****	<0.0001	****	0.0014	**
Stress vs Diet	0.4504	ns	<0.0001	****	<0.0001	****
CD						
Control vs Stress	<0.0001	****	<0.0001	****	0.1707	ns
Control vs Diet	<0.0001	****	<0.0001	****	<0.0001	****
Stress vs Diet	0.2935	ns	0.5241	ns	0.0139	*
R1						
Control vs Stress	<0.0001	****	<0.0001	****	0.0009	***
Control vs Diet	<0.0001	****	<0.0001	****	0.2813	ns
Stress vs Diet	0.2331	ns	0.3325	ns	0.0411	*
ICD						
Control vs Stress	<0.0001	****	<0.0001	****	<0.0001	****
Control vs Diet	<0.0001	****	<0.0001	****	<0.0001	****
Stress vs Diet	<0.0001	****	<0.0001	****	0.703	ns
R2						
Control vs Stress	<0.0001	****	<0.0001	****	<0.0001	****
Control vs Diet	<0.0001	****	<0.0001	****	0.0024	**
Stress vs Diet	<0.0001	****	<0.0001	****	0.2657	ns
ECD						
Control vs Stress	<0.0001	****	<0.0001	****	0.0005	***
Control vs Diet	<0.0001	****	<0.0001	****	0.0222	*
Stress vs Diet	<0.0001	****	0.0009	***	0.3142	ns
R3						
Control vs Stress	<0.0001	****	<0.0001	****	0.0069	**
Control vs Diet	<0.0001	****	<0.0001	****	0.0009	***
Stress vs Diet	0.0135	*	0.0022	**	0.8772	ns

Data are expressed as mean ± standard error; * $p < 0.1$, ** $p < 0.01$, *** $p < 0.001$, **** $p < 0.0001$, ns: no significative. SD, Simple Discrimination; CD, Compound Discrimination; R1, Reversal 1; ICD, Intradimensional shift; R2, Reversal 2; ECD, Extradimensional shift and R3, Reversal 3.

Supplementary Table S3. Multiple t-Test Comparative analysis of taxon abundance among groups

		P value between groups (t test)							
**Taxonomic rank**	**Taxa**	**MC vs MS**	**MC vs MD**	**FC vs FS**	**FC vs FD**	**MM vs MS**	**MM vs MD**	**FM vs FS**	**FM vs FD**
Phylum	Bacteroidota	↑0.8741	↑0.0805	↓**0.0003**	↓0.7300	↑0.9999	↑0.9999	↓**0.0151**	↓0.9999
	Bacillota	↓0.7762	↓0.0805	↑**0.0006**	↑0.9999	↓0.9999	↓0.9999	↑**0.0011**	↑0.9999
	Proteobacteria	↓0.9999	↑0.9999	↑0.9999	↑0.9999	↓0.9999	↑0.9999	↓0.9999	↑0.9999
Class	Bacteroidia	↑0.8741	↑0.0932	↓**0.0005**	↓0.7913	↑0.9999	↑0.9999	↓**0.0222**	↓0.9999
	Clostridia	↓0.9999	↑**0.0079**	↑0.9999	↓0.9999	↓0.1578	↓0.9999	↑**0.0015**	↑0.4355
	Bacilli	↓0.8741	↓**0.0001**	↑**0.0002**	↑0.9848	↑0.6468	↓0.9999	↑0.9999	↓0.8546
Order	Bacteroidales	↑0.8741	↑0.0903	↓**0.0005**	↓0.8168	↑0.9999	↑0.9999	↓**0.0222**	↓0.9999
	Clostridiales	↓0.9999	↑**0.0079**	↑0.9999	↓0.9999	↓0.1543	↓0.9999	↑**0.0008**	↑0.2609
	Lactobacillales	↓0.8741	↓**0.0001**	↑**0.0001**	↑0.9743	↑0.6468	↓0.9999	↑0.9999	↓0.8866
Family	Porphyromonadaceae	↑0.9995	↑0.5006	↓**0.0004**	↓0.4378	↑0.9999	↑0.9567	↓0.1901	↓0.9999
	Bacteroidaceae	↑0.9999	↑0.9999	↓0.9999	↓0.9999	↑0.9999	↑0.9999	↓0.9999	↓0.9999
	Lachnospiraceae	↓0.9999	↑0.0660	↑0.9999	↓0.9999	↓**0.0268**	↓0.9933	↑**0.0001**	↑0.3625
	Lactobacillaceae	↓0.8657	↓**0.0001**	↑**0.0001**	↑0.9614	↑0.6468	↓0.9999	↑0.9999	↓0.8809
Genus	*Odoribacter* spp.	↑0.9999	↑0.9999	↑0.9999	↑0.9093	↓0.8475	↓0.4741	↑0.9999	↑0.9944
	Alistipes spp.	↓0.9999	↑0.9999	↓0.9999	↑0.9999	↓**0.0001**	↓**0.0001**	↑0.9887	↑0.6551
	Parabacteroides spp.	↓0.9999	↑0.9999	↓0.9999	↓0.9999	0.9999	↑0.9999	↓0.9999	↓0.9999
	Barnesiella spp.	↑0.9999	↑0.9346	↓**0.0002**	↓**0.0033**	↑0.9999	↑0.5927	↓0.9999	↑0.9999
	Bacteroides spp.	↑0.8732	↑0.9999	↓0.9964	↓0.9999	↑0.6468	↑0.9999	↓0.8178	↓0.9999
	Dorea spp.	↑0.9999	↑**0.0008**	↑0.9999	↑0.9999	↑0.9999	↑**0.0001**	↑0.9999	↑0.9999
	Clostridium_XlVb spp.	↓0.9999	↓0.9999	↑0.9999	↑0.9999	↑0.9999	↑0.9999	↓0.9999	↓0.9999
	Lactobacillus spp.	↓0.2399	↓**0.0001**	↑**0.0001**	↑0.9999	↑0.9173	↓0.3995	↑0.1091	↓**0.0393**
	Helicobacter spp.	↑0.9999	↑0.9999	↑0.9999	↑0.9848	↓0.9999	↓0.9999	↑0.9999	↑0.9814

The Table showed controls (MC; FC), and control manipulation (MM; FM) versus stress (MS; FS) as well as HF diet (MD; FD) groups. MC, male control; MS, male stress; MD, male diet and MM, male manipulation; FC, female control, FS female stress; FD female diet and FM, female manipulation groups. Significant differences at $p<0.05$ were in bold.
